# Emerging Therapeutic Approaches for Cystic Fibrosis. From Gene Editing to Personalized Medicine

**DOI:** 10.3389/fphar.2019.00121

**Published:** 2019-02-27

**Authors:** Iwona Pranke, Anita Golec, Alexandre Hinzpeter, Aleksander Edelman, Isabelle Sermet-Gaudelus

**Affiliations:** ^1^INSERM U 1151, Institut Necker Enfants Malades, Université Paris Descartes, Paris, France; ^2^Centre de Référence Maladie Rare, Mucoviscidose et Maladies de CFTR, Paris, France; ^3^Faculté de Médecine, Université Paris Descartes, Paris, France

**Keywords:** cystic fibrosis, CFTR, ivacaftor, CFTR modulator, gene therapy

## Abstract

An improved understanding of the cystic fibrosis (CF) transmembrane conductance regulator (CFTR) protein structure and the consequences of *CFTR* gene mutations have allowed the development of novel therapies targeting specific defects underlying CF. Some strategies are mutation specific and have already reached clinical development; some strategies include a read-through of the specific premature termination codons (read-through therapies, nonsense mediated decay pathway inhibitors for Class I mutations); correction of CFTR folding and trafficking to the apical plasma membrane (correctors for Class II mutations); and an increase in the function of CFTR channel (potentiators therapy for Class III mutations and any mutant with a residual function located at the membrane). Other therapies that are in preclinical development are not mutation specific and include gene therapy to edit the genome and stem cell therapy to repair the airway tissue. These strategies that are directed at the basic CF defects are now revolutionizing the treatment for patients and should positively impact their survival rates.

## Introduction

Cystic fibrosis (CF) is an autosomal recessive disease that affects approximately 75,000 people in North America, Europe, and Australia alone. The life expectancy of CF patients has been constantly increasing because of symptomatic therapies. As our knowledge of the CF transmembrane conductance regulator (CFTR) structure and the functional consequences of its mutations has improved, therapeutics to restore CFTR expression and function have begun to emerge. Search for mutation-specific and mutation-independent tactics have now opened the path toward a revolutionizing approach in treating CF patients.

## Background

### CFTR Biology and Cystic Fibrosis

The CFTR is a transmembrane chloride (Cl^-^) and bicarbonate (HCO_3_^-^) ion channel that is expressed in the apical membranes of the epithelial cells of multiple exocrine organs, where it regulates salt and fluid homeostasis (Linsdell, 2014). The CFTR glycoprotein has multiple membrane-integrated subunits that form two membrane-spanning domains (MSD), two nucleotide-binding domains (NBD), and a regulatory (R) domain that acts as a phosphorylation site (Rowe et al., 2005; Riordan, 2008). MSD1 and MSD2 form the walls of the channel pore, and their conformational changes drive the channel’s opening and closing (Muallem and Vergani, 2009). Phosphorylation of the R domain, which is driven by cAMP-dependent protein kinase A and C, enhances adenosine triphosphate (ATP) association to the NBD domains and hence mediates their conformational change and their dimerization in a head-to-tail configuration. This conformation defines the open state of the channel (Riordan, 2008). Inversely, the hydrolysis of ATP drives the channel to a basal closed state (Vergani et al., 2005).

CFTR protein maturation, which is characterized by complex domain folding, assembly, and double *N*-glycosylation of the MSD2, starts early in the endoplasmic reticulum (ER) during translation and continues in the Golgi apparatus. This complex process can be mismatched and retarded at multiple steps, leading to important (60–80%) degradation of even wild-type CFTR (WT-CFTR) (Varga et al., 2004) by the ER-associated ubiquitin-dependent degradation system (Jensen et al., 1995; Gelman et al., 2002) or autophagy. After reaching the plasma membrane (PM), the WT-CFTR channel is internalized by clathrin-dependent endocytosis and recycles back to the cell surface through recycling endosomes. Because the mature WT-CFTR is very stable at the PM, a pool of 10% WT-CFTR is internalized and recycled back to the PM each minute (Sharma et al., 2004; Pranke and Sermet-Gaudelus, 2014).

Almost 2,000 mutations in the *CFTR* gene have been found to cause CF; they decrease the flow of Cl^-^ and HCO_3_^-^ through the epithelia of multiple organs, including the lung, pancreas, sweat glands, vas deferens, liver, and intestine. As a consequence, they interfere with their normal functioning. In the respiratory tracts, the lack of a CFTR function drives the accumulation of abnormally thick and sticky mucus that underlies chronic lung inflammation and recurrent bacterial infections, leading to progressive lung degradation. There is increasing evidence that airway inflammation and infection are frequently present before the appearance of symptoms; however, it is not clear which comes first. Studies on CF animal models suggest that CF causes congenital airway abnormalities, such as a narrowed trachea and that the airway surface liquid has a reduced pH level in CF, leading to an impaired bacteria-killing potential. The accumulation of mucus gives rise to sterile inflammation. These pathological conditions initiate a vicious circle that leads to bronchial wall inflammation and air trapping. The cumulation of neutrophils further enhances inflammation through the production of elastase and proinflammatory cytokines (Nichols and Chmiel, 2015). This ultimately leads to the formation of bronchiectasis. Lung disease is responsible for >95% of CF deaths (Davis et al., 1996; Stoltz et al., 2015). Currently, according to the CF foundation registry, the median survival age of those born in 2016 is predicted to be 47.7 years of age (Cystic Fibrosis Registry 2016). Because the morbidity and mortality of CF patients is mainly caused by lung disease, the principal focus of research in the CF domain and therapy development has been targeted at minimizing lung disease.

### Mutations Inducing CF

**Figure 1:**
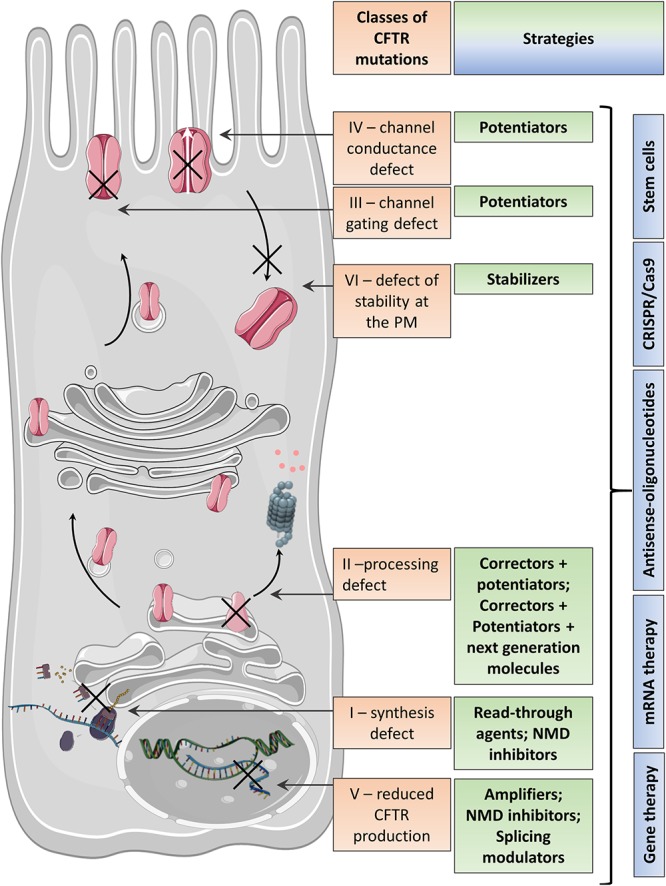
Variable CFTR protein defects causing CF disease and the corresponding therapeutic strategies.

*CFTR* mutations are divided into six classes determined by the specific defect in CFTR protein synthesis, trafficking, function, or stability (O’Sullivan and Freedman, 2009) (Figure 1 and Table 1), although many CFTR mutants present multiple defects, such as F508del-CFTR with deficient trafficking, function, and stability (Veit et al., 2016).

**Table 1:** Classification of *CFTR* mutations.

Class of mutation	CFTR defect	CFTR function	CFTR apical expression	Examples of mutations	Potential therapy	
I	Defective production	No	No	G542X, W1282X, R553X, R1162X, E822X, 1717-1G > A, 711+1G > T, 621+1G > T	Read-through agents, NMD inhibitors	

II	Impaired processing	No	No	F508del, N1303K, I507del, R1066C, S549R, G85E	Correctors, Correctors + Potentiators (C + P), C + P + next generation correctors, C + P + Amplificators, C + P + Stabilizers	Gene therapy, antisense-



III	Defective regulation	No	Yes	G551D, G178R, G551S, R560T, V520F, G970R, G1244E, G1349D	Potentiators	oligonucleotide therapy, mRNA therapy, CRISPR/Cas9, Stem cells therapy



IV	Defective conductance	Reduced	Yes	R334W, R117H, R347P, R1070W	Potentiators	

V	Reduced amount	Reduced	Reduced	3272-26A > G, 3849+10 kb C > T, A455E, D565G	NMD inhibitors, Splicing modulators, Amplifiers	

VI	Defect of stability at the PM	Reduced	Reduced	1811+1.6 kb A > G	Stabilizers	

Class I mutations lead to severely defective protein production. They are primarily nonsense or frameshift mutations introducing a premature termination codon (PTC), leading to unstable messenger RNA (mRNA) degraded by the mRNA decay pathway (NMD) (Maquat, 2004; Popp and Maquat, 2016; Martiniano et al., 2016). If the mRNA is translated, PTC decoding results in ribosome disruption and premature translation termination, usually resulting in synthesis of a non-functional, shortened CFTR protein. Large insertions/deletions and splicing mutations resulting in the absence of proteins at the PM are also included in Class I.

Class II mutations introduce defects in CFTR processing. The main example of this class is the in-frame deletion of the 508 amino acid phenylalanine (p.Phe508del, legacy name F508del) which affects about 80% of patients. Defective folding of the newly synthesized F508del-CFTR renders NBD1 instable energetically and impairs the assembly of the interface between NBD1 and MSD1/MSD2 (Du et al., 2005; Billet et al., 2013). This misfolding impairs its stability at the ER, promoting premature degradation by the ubiquitin-dependent proteasome (Lukacs and Verkman, 2012). Consequently, little or no CFTR is trafficked to the apical PM. This mutation is also associated with the impairment of Cl^-^ channel gating and decreased CFTR stability at the PM because of increased degradation by a peripheral ubiquitination-related protein quality control system (Lukacs et al., 1993).

Class III mutations produce CFTR protein localizing at the PM but with defective activation, leading to a severe decrease in the ion channel’s open probability. The c.1652G > A mutation, legacy name G551D (located in exon 11), affects about 2–4% of patients and is the most frequent in this class. In this case, the substitution of the amino acid glycine by aspartate occurs at a crucial point in the NBD1–NBD2 interface, inactivates ATP-dependent gating, and decreases open probability by ∼100-fold compared with WT-CFTR.

Class IV mutations induce channel dysfunction by defective ion conductance; these mutations mostly involve the MSD regions of the CFTR protein, forming the pore of the channel. The Class IV missense mutations provide a protein located in the apical membrane but with only the residual activity of a cAMP-dependent Cl^-^ secretion. The most common Class IV mutation is c.350G > A, legacy name R117H (located in exon 4), which affects 0.7% of patients. This substitution of arginine with histidine at position 117, which is located in the region of TMD2, reduces the channel open probability by 75%, and changes Cl^-^ and HCO_3_^-^ conductance (LaRusch et al., 2014). Because Class IV mutations lead to the biosynthesis of CFTR retaining residual function and normal regulation, simple therapies to improve their activity are efficient.

Class V mutations are characterized by reduced amounts of normally functioning CFTR at the apical PM. Most of these mutations affect pre-mRNA splicing. This induces complete or partial exclusion of the exon, generating missense, silent, or nonsense mutations and, consequently, the production of defective CFTR. Class V mutations are either intronic mutations inducing the incorporation of cryptic exons or exonic mutations altering splicing enhancer motifs. The most common mutation from this class is c.3718-2477C > T, legacy name 3849+10 kb C → T (located in intron 19), and affecting about 0.58% of patients but with a higher frequency in specific populations, such as Ashkenazi Jews.

Class VI mutations result in the decreased stability of CFTR at the apical membrane as a result of increased endocytosis or decreased recycling to the PM. An example of Class VI mutations is c.120del23. This deletion of nucleotides 120 up to 142 in exon 1 eliminates the translation initiation codon at nucleotides 133–135, and the translation instead initiates at sites in exon 4 at M150/M152/M156. This produces N-truncated proteins that are unstable and display reduced Cl^-^ channel activity (Ramalho et al., 2009).

Although mutations of Classes I–III provoke more severe CF disease with absent or very weak residual CFTR activity, mutations representing Classes IV–VI lead to relatively high residual function and are associated with milder forms of CF.

### Different Treatment Strategies

Some strategies are specific to the CFTR mutation and aim to (i) bypass a specific PTC and restore mRNA levels (read-through therapies, NMD inhibitors therapy for Class I mutations), (ii) correct CFTR folding and trafficking to the apical PM (correctors for Class II mutations), or (iii) increase the CFTR channel function (potentiators therapy for Class III mutations and any mutant with residual function located at the PM) (Figure 1 and Table 1). Other therapies in preclinical development are not mutation specific and include gene therapy to edit the genome and stem cell therapy to repair the airway tissue.

The personalization of therapy for a given patient is based on the paradigm of selecting the most effective molecule or association of molecules. The functional assays that directly or indirectly measure the CFTR activity in *in vitro* cultures of primary nasal epithelial cells (Pranke et al., 2017) and the cultures of organoids developed from intestinal epithelia (Dekkers et al., 2013, 2016) and nasal/bronchial spheroids (Brewington et al., 2018; Guimbellot et al., 2018) are promising tools to use as patient-specific biomarkers, predictive of clinical efficacy of these novel therapies.

## Genetic Therapies

Cystic fibrosis genetic therapies rely on delivering DNA or RNA, which encodes the CFTR protein or on the restoration of the *CFTR* gene (genome editing) or the CFTR mRNA (mRNA editing).

### Gene Therapy

Gene therapy implies the relocation of the proper copies of the *CFTR* gene to the epithelial cell layer in the airways with the goal of replacing the mutated gene and express functional CFTR protein. For high efficiency of this therapy, DNA coding for CFTR together with regulatory components must be adequately administered to the airways, reach the target cells, enter (transduce) the cell, and express CFTR protein. Because CF is a monogenic disease, gene therapy is particularly attractive. Despite the fact that CF is a multiorgan disease, improving respiratory manifestations will lead to a significant improvement in the patient’s quality of life and may be associated with a decrease in mortality. The inhaled route is the easiest way to access the targeted abnormal zones.

**Figure 2:**
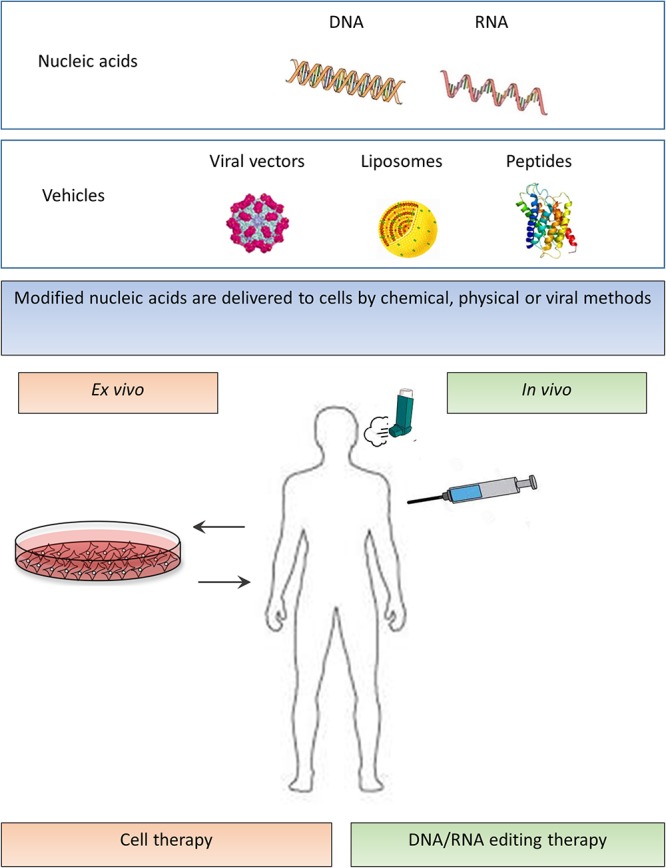
*In vivo* and *ex vivo* strategies of mutation independent approaches. Modified nucleic acids (upper panel – nucleic acids) are delivered to cells by various methods (second upper panel – vehicles). In *ex vivo* cell therapy, stem or progenitor cells are derived from the subject, and after the required modification, the cells are transferred back into the patient (lower left part of the panel). In *in vivo* DNA/RNA editing therapy, a direct transfer of genes to the patient is performed by viral or non-viral methods (lower right part of the panel).

Although gene therapy carries promise, it has several limitations. First, finding the appropriate plasmid DNA molecule model is important in terms of clinical potency (Pringle et al., 2009; Dhand, 2017). Second, natural barriers such as mucus, versatile immune responses, and intracellular limitations considerably impair gene transfer into the lungs (Osman et al., 2018). Finally, because the airway epithelium is constantly renewing, genetic therapies necessitate repeated administration. Therefore, the selection of the appropriate delivery method is essential. The most commonly used agents in gene therapy for CF are viral vectors: adenoviruses, adeno-associated viruses, and lentiviruses, but also non-viral lipoplexes and peptide nanoparticles (Figure 2).

In 1990, for the first time, Drumm et al. (1990) proved that it is possible to deliver a healthy *CFTR* gene into the adenocarcinoma cell of a CF patient by means of retrovirus transduction. Expression of a normal *CFTR* gene was linked to a cAMP-dependent Cl^-^ channel regulation in CF epithelial cells.

The first ever use of viral and subsequent non-viral gene transfer factors on nasal and bronchial epithelium was seen in clinical trials in 1993 (Zabner et al., 1993; Crystal et al., 1994; Caplen et al., 1995). Many subsequent trials have demonstrated evidence of CFTR expression but have not achieved clinical efficacy.

#### Viral Vectors—Adenoviruses

Rosenfeld et al. (1992) showed that the adenovirus-mediated transfer of DNA coding for human CFTR to a cotton-rat model by intratracheal introduction resulted in mRNA and functional protein expression. In turn, Zabner et al. (1993) performed adenoviral gene therapy tests in humans. Although neither the CFTR mRNA nor the protein were detectable after a single nasal application of the *CFTR* gene-containing vector, the nasal potential difference showed a limited improvement of the conductivity of the Cl^-^ channel. Crystal et al. (1994) was the first to detect CFTR protein in the lung and nose tissue after adenoviral vector administration. Additional surveys on gene transfer by adenovirus transduction of the nasal epithelium proved that the expression of CFTR mRNA and/or CFTR protein is transient and that the Cl^-^ transport was not fully recovered, as shown by the nasal potential difference (Hay et al., 1995; Knowles et al., 1995; Zabner et al., 1996; Bellon et al., 1997).

Despite preliminary promising data from preclinical models of nasal and pulmonary tissues (Katkin et al., 1995; Scaria et al., 1998) and a good tolerance at low-to-intermediate doses (Harvey et al., 2002), adenovirus-mediated gene transfer proved inefficient in CF patients (Joseph et al., 2001). This mostly occurred because of the lack of the coxsackie-adenovirus receptor, which is absent from the apical surface of most human airway epithelial cells (HAE) (Walters et al., 1999). The transduction efficiency was increased by a tight junction opener (Gregory et al., 2003) and the association of the adenovirus to the 2-(diethylamino) ethyl ether (DEAE) dextran. However, clinical use of these tight junction openers could introduce the risk of systemic invasion when considering the significant presence of bacteria in the CF lung.

#### Adeno-Associated Virus (AAV) Vectors

The substantial achievement in gene therapy by using rAAV for congenital blindness, hemophilia B, and lipoprotein lipase deficiency boosted scientists’ interest in more carefully investigating a possible rAAV-based gene therapy approach for CF (Vidović et al., 2015). Here, rAAV is a derivate of wild-type AAV and is not related to human pathology; this vector holds promise because it is perceived to be a safe vector due to its low immunogenicity, lack of viral genes, and non-integrating character (Dismuke et al., 2014). Vidović et al. (2015) showed that the rAAV-mediated gene delivery of a shortened R domain deleted-CFTR led to the correction of the CF phenotype in CF mice nasal mucosa and in the intestinal organoids derived from CF patients. Scientists must now focus on methods to enlarge AAV tropism and to diminish its immunogenicity while improving CFTR expression and perseverance in the lungs. The efforts to improve the AAV vector efficacy in significant animal models of CF and the confirmation of the potent transduction of human epithelia for therapeutic use still remain challenging, but not unreachable (Hart and Harrison, 2017).

Another way to tackle packaging restraints and expand AAV tropism is the use of human Bocavirus-Type-1 (HBoV1) with AAV2 genome. Like AAV, the HBoV1 is a parvovirus that exhibits a high level of tropism for both transduced human cells polarized in the air–liquid interface cultures and for the apical membrane of the human airway epithelium. HBoV1 possesses a bigger capsid, allowing for the packing capacity of 5543 nt compared with an AAV of 4679 nt. The chosen animal model for exploring therapeutic approaches with rAAV2/HBoV1 vector has been CF ferrets because these animals mimic very well the physiological aspects of CF lung disease. The study by Yan et al. (2017) confirmed that *in vitro* and *in vivo* ferret epithelium is susceptible to transduction by the rAAV2/HBoV1 vector. Moreover, the experiments showed that repetitive dosing *in vivo* was efficient in sustaining transgene expression (Yan et al., 2017).

#### Non-viral Vectors

The anxiety concerning unwanted immunogenic reactions, possible transgene miss-insertions, difficulty in packing a nucleic acid of an excessive size, and issues in bulk-production have shed light on non-viral vector alternatives (Foldvari et al., 2016). Advancements in liposomal vectors have demonstrated the secured and feasible delivery of bulky DNA molecules (Foldvari et al., 2016; Hart and Harrison, 2017). A randomized, double-blind, placebo-controlled Phase 2b trial carried out in the United Kingdom with a repeated nebulization of non-viral *CFTR* gene showed a modest improvement in FEV_1_ (forced expiratory volume in 1 s) compared with placebo at 1 year, demonstrating the stabilization of the lung function in treated patients (Alton et al., 2015). The conclusion is speculative because the difference was mainly a consequence of decrease in the placebo group. Moreover, there was no evidence of WT-CFTR expression in respiratory cells (Mottais et al., 2017). Although disappointing, this trial demonstrated that dosing repetition is harmless, so there is a necessity to improve nucleic acid delivery to the target cell (Hart and Harrison, 2017).

To protect the DNA from extracellular factors such as mucus, bacteria, or inflammation and physical deterioration during inhalation, DNA nanoparticles can be shielded by biodegradable poly(b-amino esters) (PBAEs) polymers with a thick sheet of polyethylene glycol (Mastorakos et al., 2015). Furthermore, a cationic lipid labeled GL67A38 has shown endosomal discharge of plasmid DNA and stabilization during aerosol implementation although transfection efficiency was low (Dhand, 2017). Recent preclinical work has also demonstrated the ability of transferring circular pieces of DNA that retain transgene and regulatory elements by nanoparticles (Hart and Harrison, 2017). The use of synthetic vectors may also be considered when trying to decrease immunogenicity and improve integration (Lentz et al., 2005; Catanese et al., 2012).

### CRISPR/Cas9 Approach

**Figure 3:**
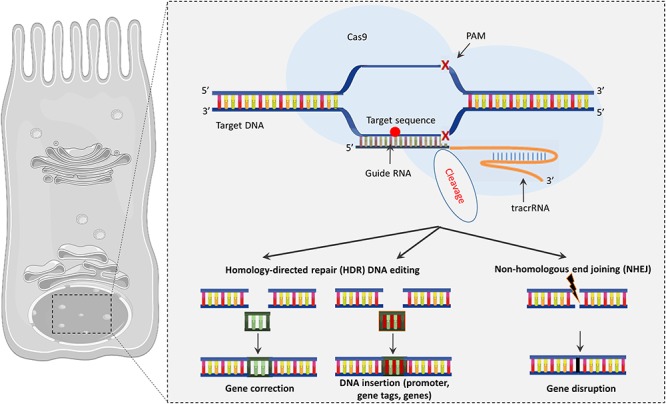
The CRISPR/Cas9 system. Cas9 endonuclease binds to the target site using a guide RNA to precisely cut DNA allowing genome editing. CRISPR/Cas9 may drive to gene correction or DNA insertion through HDR or modify the sequence through NHEJ, which results in insertions/deletions.

The CRISPR (clustered regularly interspaced short palindromic repeats)/Cas9 approach is a gene-editing strategy in which the specific mutated sequence of the defective *CFTR* gene is corrected by changes introduced into the DNA (Figure 3). CRISPR/Cas9 technology has been developed based on the bacterial defense mechanisms against “foreign” DNA (e.g., virus) (Alapati and Morrisey, 2017). In this mechanism, “foreign” DNA incorporates multiple small pieces into a locus consisting of short palindromic repeats, called CRISPR. Upon re-exposure to the introduced DNA, the CRISPR locus is transcribed into small RNAs that lead the Cas9 endonuclease to a particular spot in the added DNA, based on the DNA–RNA sequence complementarity, which creates a double-stranded opening and protects the host bacterium. CRISPR/Cas9 technology uses a protein-RNA complex composed of an enzyme—Cas9 endonuclease bound to a guide RNA (gRNA) molecule. Engineered Cas9 (Type II bacterial endonuclease) cleaves the DNA in a sequence-specific mode defined by the gRNA component that recognizes through the complementarity the mutated sequence and creates a specific double-stranded break (Figure 3). The cell can then fill the excised portion with the correct gene sequence through homologous directed repair (HDR), which is the desirable plan of action but can fail occasionally as in the case of the non-homologous end joining (NHEJ), which results in insertions/deletions formation. CRISPR has been recognized as the most powerful gene-editing tool when compared with zinc-finger (ZFN) and transcription activator-like effector (TALEN) endonucleases. The designed gRNA has an identical sequence as the desired site in the genome, which allows for intervention at the level of the DNA sequence with high precision, acting with molecular “scissors” to cut the DNA at the desired point and replace it with the correct sequence (White et al., 2017).

The two main benefits of this precision-made correction of the faulty gene are as follows: (i) the modified gene prevails under control of its endogenous promoter, allowing life-long expression and natural adjustment in the cell; (ii) gene repair has the ability to bypass the engagement of the external DNA, hence decreasing the chance of insertional mutagenesis.

Non-viral (lipidic or polymeric) vectors seem suitable for achieving CRISPR/Cas9 expression probable transgene integration or secondary tumor initiation (Li et al., 2015). These vectors also prevent immune responses, which has been observed in the case of viral vectors, and limit off-target activity. The optimal mode of CRISPR/Cas9 introduction into the lungs is aerosol delivery devices combined with nanoparticle suspensions. Nevertheless, inhaled therapy can become entrapped and unable to cross the dense and viscous pathological mucus layer. Despite the fact that the CRISPR approach is in its early beginnings, it presents possible significant outcomes for future CF therapy benefits.

The first studies on CRISPR/Cas9 to develop a potential therapy for CF were published in 2013. Schwank et al. (2013) tested the recovery of a functional CFTR protein in intestinal organoids obtained from CF pediatric patients carrying the F508del mutation. After lipofectamine-mediated transfection of intestinal stem cells, CRISPR/Cas9 gene editing repaired the mutation at the *CFTR* locus through the *CFTR* gene substitution approach (HDR), improving the forskolin-induced response and prompting organoid swelling. The same group suggested colonic transplantation of genetically corrected organoids as a probable perspective (Yui et al., 2012). Another successful delivery of CRISPR/Cas9 was reported by Bellec et al. (2015). They knocked-down the *CFTR* gene in HAE cells and Calu-3 cells using the CRISPR/Cas9 approach delivered with HIV-1 lentivirus. CRISPR/Cas9-directed gene modification was associated with a decline of transepithelial Cl^-^ secretion and a decrease in response to a CFTR inhibitor, as measured in polarized cell cultures in Ussing chambers.

Two other groups showed gene correction in CF-specific iPSCs (induced pluripotent stem cells) with the use of gene editing (Firth et al., 2015; Crane et al., 2015). Firth et al. (2015) used CRISPR technology and a piggyBac-based donor to obtain footprint-free gene correction at the *CFTR* locus in patient-derived iPSCs. The recovery of expression and function of CFTR, as measured by patch-clamp, in lung epithelial cells that were differentiated from edited iPSCs was demonstrated (Firth et al., 2015). Crane et al. (2015) used ZFN technology, which is less efficient than CRISPR, to correct the *CFTR* gene. When the repaired CF-iPSCs were differentiated into lung epithelial cells, a mature CFTR glycoprotein was expressed, which in turn recovered CFTR Cl^-^ channel activity (Crane et al., 2015).

Sanz et al. (2017) investigated the application of a CRISPR/Cas9-based NHEJ method to edit a small number of CF-causing mutations: c.1679 + 1634A > G, c.3140-26A > G, and c.3718-2477C > T CF, which create alternative splice sites that produce pseudo-exons or extend existing exons. The group demonstrated that CRISPR Cas9/gRNA pairs are useful for successful excision via a NHEJ pathway. NHEJ-mediated excision took place in ≥25% of transfected cells, a degree of editing that is 10-fold higher than their previous study of homology directed repair gene editing, with Cas9/gRNA in the same locus in the same cells (Hollywood et al., 2016).

### Antisense-Oligonucleotide-Mediated Therapy

Antisense oligonucleotides (ASOs) are single-stranded synthetic RNA-like molecules that can selectively change gene expression by means of various techniques regulated by their chemistry and antisense oligomer design. Because these mechanisms of action are based on the complementary base pairing to the target sequences, oligonucleotides are highly specific compounds. Antisense oligonucleotides are being chemically modified to improve cellular uptake and intracellular stability and to decrease cell toxicity. From a pharmacological point of view, they are attractive forms of medication because they are resistant to nucleases and have good pharmacokinetic properties.

Zamecnik et al. (2004) constructed a modified oligodeoxyribonucleotide to replace the three missing bases caused by F508del mutation in the CFTR mRNA. This treatment *in vitro* showed improved CFTR function (Zamecnik et al., 2004). The company ProQR employs single-stranded antisense RNA-based oligonucleotides that encompass the lacking bases and behave as guide sequences to restore the targeted abnormal mRNA in cells carrying the F508del mutation. Phase II clinical trials with this modified RNA oligonucleotide QR-010 (Eluforsen), which is dedicated to F508del mutation, showed increased CFTR function in patients’ nasal mucosa, good drug tolerance, and improved quality of life.

Antisense oligonucleotides can repair abnormal mRNA or alternatively target an RNA transcript for degradation through RNaseH activation. Recently, Crosby et al. (2017) tested ENaC-specific antisense oligonucleotides delivered by inhalation in mouse models for the prevention and reversal of lung symptoms in CF. Aerosol-delivered ENaC ASOs down-regulated ENaC and mucus marker expression, ameliorated goblet cell metaplasia, inflammation status, and airway hyper-responsiveness (Crosby et al., 2017).

Most of the efforts in antisense oligonucleotides research focus on splicing alteration to skip an exon enclosing a nonsense or frameshift mutation or alternatively recover the reading frame, expecting here that the end isoform will retain improved function compared with the mutated protein. ASOs have also been shown to modulate splicing in cells with the CFTR splicing mutation c.2657+5G > A, which causes exon 16 to be omitted along the splicing (Igreja et al., 2016). Single-stranded DNA oligonucleotides of 19 nucleotides having 2′-*O*-methyl modified ribose and a phosphorothioate backbone were modeled to hybridize to pre-mRNA and correct aberrant splicing in HEK293 cells expressing the c.2657+5G > A mutant *CFTR* minigene (Igreja et al., 2016). A similar correction was achieved for 3849 + 10 kb C → T, a mutation identified in 5% of Ashkenazi Jewish patients, which constitutes a novel donor site in intron 19, causing an 84 base-pair pseudo-exon to be incorporated into the mRNA, and generating a downstream PTC (Friedman et al., 1999).

### mRNA-Mediated Therapy

Whereas conventional gene therapy targets the nucleus, in mRNA therapy, a right nucleotide sequence coding for CFTR is targeted at the cell cytoplasm. In both cases, a normal protein is produced, though the concepts based on mRNA delivery are more convincing because they are not required to overcome the nuclear membrane barrier. Apart from this, chemically changed mRNA displays various benefits over other nucleic acids. The most valuable characteristics are a transient protein expression, decreased immunogenicity, superior translation efficacy, enhanced stability, and pharmaceutical safety because modified mRNA is not incorporated into the host genome (Kuhn et al., 2012). The mRNA can be delivered to the cell by using liposomal or polymeric non-viral vector formulations that are administered via several routes, for example, intraperitoneally, intravenously, or intratracheally.

The transfection of chemically modified WT-CFTR mRNA in CFBE41o-cells homozygous of the F508del mutation re-established cAMP-induced CFTR currents (measured in Ussing chambers) similar to WT cells as a result of the mRNA-driven replacement of functional channels (Bangel-Ruland et al., 2013). Immunofluorescence and biochemical approaches have confirmed the expression of apically located WT-CFTR after optimized WT-CFTR mRNA transfection. Primary cultured human nasal epithelial cells were characterized by nearly a twofold improvement in the cAMP-stimulated CFTR current after mRNA transfection.

Robinson et al. (2018) used a clinically relevant lipid-based nanoparticle (LNP) for the packaging and transport of large chemically modified CFTR mRNA (cmCFTR) to bronchial epithelial cells derived from patients. The experiments showed an increase in membrane-localized CFTR and rescue of its primary function as a Cl^-^ channel. Nasal application of LNP-cmCFTR recovered CFTR-mediated Cl^-^ secretion to conductive airway epithelia in CFTR knockout mice for at least 14 days. The CFTR activity peaked on the 3rd day post-transfection, retrieving up to 55% of the net Cl^-^ efflux that is distinctive for the healthy mice (Robinson et al., 2018).

## Stem Cell Therapy

The biggest obstacle to overcome in regenerative medicine is to determine the relevant cells that will be capable of repairing a defect. The desired cell should be non-immunogenic, patient specific, easy to make proliferate, and easy to modify (Guo et al., 2017). Because endogenous progenitor cells are difficult to recognize and insufficient in their quantity and activity, focus has been directed on exogenous cell sources (McQualter et al., 2010; Rock and Königshoff, 2012). The discovery of iPSCs inspired a discussion about whether iPS cells are a copy of embryonic stem cells (ESCs). Yamanaka’s group first obtained the iPS cells by reprogramming mouse fibroblasts and next confirmed the results with the use of human fibroblasts (Takahashi et al., 2007). Nowadays, the existing therapeutic probability of ESCs is even more alluring when combined with the CIRSPR/Cas9 approach (Wagner et al., 2016). Indeed, repairing a CFTR mutation has been demonstrated in human iPSC cells by means of CRISPR/Cas9. Skin fibroblasts derived from CF individuals were reprogrammed toward iPSCs, transfected with the CFTR/Cas9 gRNA vector, corrected to their WT phenotype, and further entirely differentiated into cells of proximal and distal airways (Crane et al., 2015; Pollard and Pollard, 2018). This suggests that theoretically, stem lung cells restored to their WT phenotype could be regrafted to lung niches to further redifferentiate them into respiratory cells.

Moreover, the CRISPR/Cas9 method showed the total recovery of CFTR protein function in organoids—intestinal stem cells placed in culture—derived from children with CF (Schwank et al., 2013). The possibility to engraft cultured colon organoids was tested in a mouse by using effective transplantation into a superficially damaged mouse colon. The integration of grafted cells into the mouse colon resulted in a coating with epithelium in the deprived area of the damaged colon (Yui et al., 2012; Firth et al., 2015). However, this technology is time- and labor-intensive and challenging in terms of obtaining entirely differentiated lung-specific cell subsets (Wagner et al., 2016).

Presumably, ESCs/iPSCs assays will become a potent method to better forecast patients’ clinical responses to CFTR modulators. The creation of an array of iPS cell lineages possessing the characteristics of different CF mutations will provide a powerful tool for selecting the potential drug to repair functional deficiencies (Schmidt et al., 2016; Simsek et al., 2016; Conese et al., 2018). Importantly, safety concerns about ESC- and iPSC-based cell grafts, including the transmission of possible genetic abnormalities and tumor risk, may hamper potential clinical therapeutic purpose. A clear answer is needed to define if the mutations were already pre-existing in the cells of origin or if they were introduced during the reprogramming process. Future preclinical risk assessments need to better establish tumor and disease risk related to the therapeutic use of iPSC derivatives (Martin, 2017).

## Read-Through Therapies and NMD Inhibitors

Read-through agents (Table 2) are directed at in-frame nonsense mutations. Some PTCs are more “permissive” than others to NMD, leading to residual levels of mRNA. This residual level of mRNA is the target for “read-through” agents, whose goal is to reduce the ribosomal ability to proofread and enable ribosomes to skip the PTC, leading to the formation of functional protein. Several factors are challenging when it comes to read-through treatment efficacy: (i) the level of drug-induced read-through, (ii) the amount of target transcripts, and (iii) the activity of the recoded protein (Pranke et al., 2018).

**Table 2:** Strategies of treatment for personalized CF medication and compounds tested pre-clinically and clinically.

Therapy		Compounds
Read-through agents	Pre-clinical	Geneticin, RCT101
	
	Clinical	Gentamicin ↓, NB124 (ongoing), PTC124 ↓
	
Potentiators	Pre-clinical	ATP analogs (P-ATP, 2′-dATP, P-dATP), IBMX, PG-01, VRT-532, dihydropyridine blockers of L-type calcium channels, CO-068, CB subunit of crotoxin, P5, G01, A01, A02, H01, H02, H03
	
	Clinical	Genistein ↓, curcumin ↓, VX-770 ↑, PTI-808 (ongoing in triple combination), GLPG1837 (ongoing)

Correctors	Pre-clinical	Curcumin, HDAC inhibitors (SAHA), Corr-4a, VRT-325, glafenine, RDR1, 407882, FDL169,
	
	Clinical	4PBA ↓, miglustat ↓, sildenafil ↓, VX-809 (Orkambi^®^) ↑, VX-661 (Symdeko^®^) ↑, VX-440, VX-152, VX-659, VX-445 (ongoing in triple combinations), cavosonstat (ongoing), GLPG2222, GLPG2851, GLPG2737, GLPG3221 (ongoing in triple combinations), PTI-801 (ongoing in triple combination)

Amplifiers	Clinical	PTI-428 (ongoing in triple combination)

Stabilizers	Pre-clinical	HGF (hepatocytes growth factor), VIP (vasoactive intestinal peptide)
	
	Clinical	Cavosonstat

↑-*indicates succesfull clinical outcome and the FDA approval, ↓-indicates unsuccesfull clinical attempt.*

Aminoglycosides were the first read-through agents tested for CF disease (Howard et al., 1996). Aminoglycoside antibiotics are amino sugars that interact with the ribosome at the A-site and imitate a conformational change in the ribosomal RNA that normally is induced by codon–anticodon pairing; therefore, this promotes near-cognate tRNA incorporation and increases the number of PTC misreading, allowing translation to continue to the correct termination codon. Gentamicin and geneticin present read-through potential (Wilschanski et al., 2000; Wilschanski et al., 2003; Kandasamy et al., 2012). Gentamicin was found to display beneficial effects in patients with at least one Class I mutation, as assessed by an improvement in nasal potential difference after topical nasal application (Wilschanski et al., 2003) and intravenous administration (Sermet-Gaudelus et al., 2007). These small-scale clinical trials provided a proof-of-concept for read-through efficiency. However, gentamicin and geneticin cannot be used in clinics because of serious renal toxicity and ototoxicity. Several studies have been performed to develop chemically modified aminoglycosides to provide higher activity and less toxicity (Nudelman et al., 2009; Rowe et al., 2011; Xue et al., 2014). NB124 (ELX-02, ELOXX Pharmaceuticals) is a derivative of aminoglycoside that is modified to provide a higher level of read-through activity than gentamicin (Xue et al., 2014). It has been shown that NB124 restores CFTR function to roughly 7% of WT levels. Its read-through potency has been shown in respiratory cell lines for the most prevalent PTCs—G542X, R553X, R1162X, and W1282X—and in primary human bronchial epithelial (HBE) cells from patients carrying the G542X mutation. Moreover, tests of ototoxicity in the tissue-based model showed that this compound is also less cytotoxic than gentamicin. Lower levels of toxicity and a higher level of PTC suppression by NB124 are achieved through a strong preference for cytosolic versus mitochondrial ribosomes. A Phase II clinical trial will begin in 2019 in Belgium.

High-throughput screening (HTS) identified PTC124 (Ataluren^®^, Translarna^TM^ PTC Therapeutics) (Welch et al., 2007). The systemic administration of PTC124 in CF mice expressing a human CFTR-G542X transgene induced CFTR function rescue. This small molecule was tested in a Phase II clinical study (Kerem et al., 2008; Sermet-Gaudelus et al., 2010) with contradictory results. A first Phase III trial did not deliver any significant changes in FEV_1_ although there was a positive trend favoring Ataluren^®^(-2.5% change) over the placebo (-5.5% change; *p* = 0.124) (Wilschanski et al., 2011). New clinical placebo-controlled Phase III trials excluding inhaled tobramycin, which could antagonize the effect that PTC124 has on the ribosome, demonstrated no evidence in improving FEV_1_, nor any benefit in bronchial exacerbations. The clinical development of the compound is now stopped in the field of CF (Kerem et al., 2014).

Other factors may also influence the read-through efficiency, for example the PTC identity, the neighboring mRNA sequence, the NMD efficiency controlling the level of mRNA, the geometry of the tRNA-mRNA complex in the presence of the drug at the ribosome-decoding center, and the function of the protein, which is neoformed.

Therefore, new tracks of research for PTC-associated mutations are now being explored, including (i) NMD inhibitors, (ii) the association with CFTR modulators to improve CFTR expression/function of the neoformed protein, and (iii) factors that force mismatched base pairs to adopt a Watson–Crick geometry.

ReCode Therapeutics has been developed as an innovative therapeutic approach that utilizes suppressor transfer RNA (tRNAs) with the goal of correcting CF-causing nonsense mutations. RCT101 is a therapeutic nucleic acid actively studied in preclinical models. This modified tRNA is transported to cells by patented nanoparticles to correctly “recode” the translating protein. The unpublished data from experiments on human HBEs from patients with genotypes G542X/G542X and F508del/G542X showed that RCT101 significantly increased CFTR-dependent Cl^-^ secretion as a single active agent or in combination with VX-809 and VX-770 (Hagemeijer et al., 2018).

## CFTR Modulator Therapies

A CFTR modulator is a pharmaceutical agent that targets a specific defect in the CFTR protein that is caused by mutation in the *CFTR* gene. This modulator does not correct mutations in the gene but rather targets the errors that occur post-transcriptionally, either during protein folding, trafficking up to the PM, or CFTR functioning.

The CFTR modulators are classified into four main groups: potentiators, correctors, amplifiers, and stabilizers (Table 2). They are different in their mechanisms of action, which can be determined by the type of protein defect they target (Sloane and Rowe, 2010).

### Potentiators

Therapeutic agents that improve the channel-open probability and potentiate mutated CFTR gating are called potentiators. A large number of proof-of-concept studies have been published to demonstrate that ATP analogs and small-molecule agents have good potentiation activity. Chemically modified analogs of ATP have demonstrated a significant increase in the open probability of CFTR, with P-ATP [*N*^6^-(2-phenylethyl)-ATP] having the highest affinity and efficacy (Zhou et al., 2005; Bompadre et al., 2008). It has been demonstrated that P-ATP increases the open probability of G551D-CFTR and F508del-CFTR (Miki et al., 2010). Another ATP analog 2’-dATP (2′-deoxy-ATP) also enhances the gating of WT-CFTR and G551D-CFTR (Aleksandrov et al., 2002; Cai et al., 2006). Remarkably, both modifications in ATP have synergic effects in the potentiating gating of G551D- and F508del-CFTR (Miki et al., 2010). Nevertheless, issues with bioavailability and potential unspecific binding to other proteins involved in multiple physiological functions limit the utility of ATP analogs in clinics.

The agents that increase the intracellular concentration of cAMP and hence amplify PKA activity and the phosphorylation level of the R domain might also improve the activity of defective CFTR. First, Drumm et al. (1991) found that IBMX (3-isobutyl-1-methylxanthine), a compound that increases intracellular cAMP by inhibiting phosphodiesterase, increases PKA activity and makes F508del-CFTR more responsive to activation. Second, an isoflavone derivative genistein is a tyrosine kinase inhibitor that can also activate CFTR in intact cells (Illek et al., 1995, 1996). Illek et al., (1995 1996) proposed that this effect was mediated by increasing intracellular cAMP; however, other independent studies have demonstrated that genistein directly targets and binds to the CFTR protein (French et al., 1997; Hwang et al., 1997; Weinreich et al., 1997). Genistein entered the clinical study as a duo therapy with phenylbutyrate; however, the results were not satisfying. Other flavonoids (apigenin, kaempferol, and quercetin) have been found to enhance the currents *in vitro* and *in vivo* by increasing the CFTR channel’s open probability (Illek and Fischer, 1998). Naturally existing curcumin also extends the channel opening duration of WT-CFTR (Berger et al., 2005), F508del-CFTR (Berger et al., 2005), and G551D-CFTR (Wang et al., 2007) although data from clinical investigations have not been conclusive. Similarly, it was proposed that sildenafil, a phosphodiesterase (PDE5) inhibitor, activates the guanylate cyclase and increases the intracellular cGMP and hence CFTR activity (Lubamba et al., 2008).

First cell-based fluorescence HTSs by Verkman’s laboratory (Yang et al., 2003; Pedemonte et al., 2005b) and Vertex Pharmaceuticals (Van Goor et al., 2006), applying a Fischer rat thyroid (FRT) cell line stably expressing F508del-CFTR and an YFP sensitive to iodide, led to the discovery of several classes of small-molecule potentiators (Yang et al., 2003). Screening realized by Pedemonte et al. (2005b) showed the ability of the phenylglycine molecule PG-01 to restore the opening of F508del-CFTR almost to the level of WT-CFTR. Van Goor et al. (2006) identified VRT-532 (pyrazole). As shown by Pasyk et al. (2009) and Wellhauser et al. (2009), VRT-532 potentiates the gating of G551D- or F508del-CFTR through direct interaction with CFTR and the restoration of its defective ATPase activity (Pyle et al., 2011).

In 2009, Vertex Pharmaceuticals discovered VX-770 (Ivacaftor), which potentiates CFTR activity. First, they showed that VX-770 increases the activity of F508del- and G551D-CFTR using the patch-clamp technique and Cl^-^ secretion measures in bronchial epithelial cell cultures sampled from patients carrying these mutations (Goor et al., 2009). VX-770 prolongs the opening duration of WT-CFTR (Hwang and Sheppard, 2009), acting independently of ATP hydrolysis and NBD domain dimerization, because VX-770 efficiently potentiates G551D-CFTR (Van Goor et al., 2009; Eckford et al., 2012; Jih and Hwang, 2013), E1371S-CFTR (Jih and Hwang, 2013), and WT-CFTR in the absence of ATP (Eckford et al., 2012; Jih and Hwang, 2013). Eckford et al. (2012) suggested that VX-770 interacts directly with CFTR and induces an unconventional mode of gating. Although VX-770 increased the residual forskolin-stimulated channel activity in HBE cell cultures from some F508del-homozygous patients (Van Goor et al., 2009), a Phase II investigation of F508del-homozygous patients showed no improvement in FEV_1_ (Flume et al., 2012).

A Phase II and Phase III study in patients carrying the G551D mutation showed that ivacaftor efficiently improved predicted FEV_1_ in as early as 15 days, reaching a 10.6% increase in FEV_1_ at 24 weeks of treatment (*p* < 0.001). Ivacaftor decreased sweat chloride concentration by 48 mmol/l compared with the placebo (*p* < 0.001), reduced the frequency of pulmonary exacerbations by 55% (*p* = 0.001), and increased the weight of patients to 2.7 kg (*p* < 0.001) (Ramsey et al., 2011). Phase II trials also showed significant improvements of the channel function in the nasal and sweat gland epithelia (within-subject) (Accurso et al., 2010). Children with “silent lung disease,” which is characterized by normal initial FEV_1_, also demonstrated a significant improvement in FEV_1_ and lung clearance index. These results demonstrated that correcting CFTR at the molecular level can translate into outstanding clinical improvements. VX-770 (trade name Kalydeco^®^) became the first CFTR modulator approved for use in clinics and was initially approved in the United States (beginning of 2012), then in Europe and Canada (end of 2012), Australia, and New Zealand (2013).

Further development of ivacaftor demonstrated its clinical benefit in eight additional Class III gating mutations, including S549N and G551S, confirming improvement in lung function, BMI, sweat chloride, and CFQ-R; in addition, this method did not have safety concerns (De Boeck et al., 2014). Ivacaftor proved effective in a preschool population in open-label studies, highlighting an increase in fecal elastase and potential reversal in early pancreatic insufficiency status. An important concern was abnormalities of liver function tests in this population. Finally, ivacaftor demonstrated substantial activity in the non-gating mutation R117H, with amelioration in sweat chloride and CFQ-R scores in all age groups, whereas respiratory improvement was significant only in adults, perhaps because of the disease being more established in these patients (Yu et al., 2012; Carter et al., 2015).

Given these outstanding clinical benefits, the Food and Drug Administration (FDA) approved ivacaftor for marketing authorization based on *in vitro* assays for a number of mutations with residual function. Nowadays, ivacaftor is approved by the FDA for patients aged 1 and older who have one of the following gating mutations: G178R, S549N, S549R, G551D, G551S, G1244E, S1251N, S1255P, or G1349D; one of the following residual function mutations: A455E, E193K, R117C, A1067T, F1052V, R347H, D110E, D110H, F1074L, R352Q, G1069R, R1070Q, D579G, K1060T, R1070W, D1152H, L206W, S945L, D1270N, P67L, S977F, E56K, or R74W; one of the following splice mutations: 711+3A→G, 3272-26A→G, E831X, 2789+5G→A, or 3849+10 kb C→T; or the conduction mutation R117H.

Since the approval of ivacaftor, many other potentiators have been found. A number of these are still in preclinical development. A screen of the approved drugs performed by Galietta’s laboratory picked out dihydropyridine blockers of L-type calcium channels to have the potentiation activity of F508del-CFTR (Budriesi et al., 2011); however, their clinical usefulness is unclear because of side effects (e.g., off-target cardiac effect). In turn, Faure et al. (2016) showed that a CB subunit of crotoxin from *Crotalus durissus terrificus* interacts with the NBD1 domain of both WT- and F508del-CFTR and increases their Cl^-^ channel currents. To identify the potentiators that act synergistically with correctors, Verkman’s laboratory screened the analogs of previously found P5 potentiators and unrelated synthetic small molecules. They found 12 of the most active compounds, including a thiophene (G01), a 2-thioxo-4-amino-thiazoles (A01 and A02), and pyrazole-pyrrole isoxazoles (H01, H02, and H03), with a higher potentiating efficacy in FRT cells than VX-770.

An investigational CFTR potentiator proposed by Proteostasis Therapeutics—PTI-808—was found to enhance the function of F508del-CFTR (2018 ECFS Conference, New Frontiers in Basic Science of Cystic Fibrosis) and is currently in Phase I clinical trials together with PTI-801 and PTI-428 as a combination therapy.

Interestingly, dihydro-5H-thieno[2,3-c]pyran-2-yl)-1H-pyrazole-3-carboxamide) GLPG1837, a more recent potentiator developed by Galapagos, exhibits a higher efficacy than VX-770 for G551D-CFTR (Van der Plas et al., 2018). Similar to VX-770, GLPG1837’s underlying mechanism is independent of NBD domain dimerization and ATP hydrolysis. By applying GLPG1837 with VX-770 together, Yeh et al. (2017) provided evidence that these two molecules probably act in competition for the same site of action, whereas GLPG1837 and the ATP analog P-dATP work synergistically through two different sites. Two Phase II clinical studies are now conducting enrollment to test the GLPG1837 compound in patients with a S1251N mutation and G551D mutation.

### Correctors

The CFTR correctors are small molecules that improve the trafficking of mutated CFTR (Class II mutations, e.g., F508del) from ER to the apical PM and increase CFTR cell surface expression. These correctors improve defective CFTR folding and cellular processing by direct binding (called pharmacological chaperones) or modulate protein homeostasis and the quality control system of the cell to modify the recognition and processing of misfolded CFTR (called proteostasis regulators). Because F508del-CFTR presents multiple defects, the development of correctors is a greater challenge than the development of potentiators (Thibodeau et al., 2010). Indeed, correction of F508del-CFTR requires the following: (i) rescue to native folding by the restoration of NBD1 energetics and interface instability; (ii) evasion of the protein from ER quality control; (iii) enhancement in the apical cell membrane localization; and (iv) improvement in CFTR-dependent Cl^-^ secretion. Hence, a strategy to combine the correctors with potentiators and even an amplifier or stabilizer into a “combination therapy” was tested *in vitro* and in clinical trials.

The initial studies focused on the regulation of proteostasis for F508del-CFTR. Early studies have shown that some drugs approved for other diseases have the corrector activity for F508del-CFTR. The compound 4-phenylbutryate (Buphenyl, 4PBA), a chemical chaperone that stabilizes the folding of proteins, has been found to increase F508del-CFTR PM expression in cell culture models (Rubenstein and Zeitlin, 2000). However, this clinical trial failed to confirm the correction activity, as measured by nasal potential difference (Zeitlin et al., 2002). Curcumin (Egan et al., 2004; Lipecka et al., 2006) blocks calcium from entering into the ER and thus may interfere with the calcium-dependent chaperones that are involved in the degradation of the CFTR. Initial tests in patients failed to confirm any efficacy. Miglustat (n-butyldeoxynojyrimicin) is an alpha-glucosidase inhibitor that may interfere with F508del-CFTR misfolding quality control (Noël et al., 2008; Robert et al., 2008; Norez et al., 2009). Although Miglustat efficiently corrected the functional cell surface expression of F508del-CFTR in cell culture models and mice, a small clinical study did not confirm the corrector activity. Wang et al. (2006) demonstrated that increased PM localization of F508del-CFTR can be achieved by the down-regulation of Aha1 (an Hsp90 cochaperone), whereas Hutt et al. (2010) proposed that the inhibition of histone deacetylase HDAC7 activity with HDAC inhibitors (Suberoylanilide Hydroxamic Acid, SAHA) facilitates F508del-CFTR folding and stability and corrects F508del-CFTR. However, a later study by Bergougnoux et al. (2017) had contradictory conclusions, with experiments in human nasal epithelial (HNE) cells showing that SAHA decreased CFTR transcript and protein levels.

A second strategy was based on the HTSs of small molecules. The first corrector identified with this methodology was bithiazole Corr-4a (bisaminomethylbithiazole, C4) (Pedemonte et al., 2005a), whose later analogs have improved potency (Yu et al., 2008). Corr-4a stabilizes both ER- and PM-localized F508del-CFTR. This improves the domain assembly (Loo et al., 2008, 2009) rather than NBD1 stability (Farinha et al., 2013; Okiyoneda et al., 2013). HTSs by Vertex Pharmaceuticals introduced new correctors (Van Goor et al., 2006), including VRT-325 (quinazolinone, C3), which stabilizes both the ER- and PM-localized F508del-CFTR by improving domain assembly (Loo et al., 2008, 2009). Both Corr-4a and VRT-325 may not function through direct binding to CFTR because they are not specific to CFTR (Van Goor et al., 2006, 2011). Although Corr-4a and VRT-325 present a correction activity *in vitro*, they did not find a pharmacological use because of the high toxic effects and low *in vivo* efficacy.

Other small-scale screenings have provided further correctors, such as the drug glafenine (Robert et al., 2010), phenylhydrazone RDR1 (Sampson et al., 2011), and candidate molecules from computational screening (Kalid et al., 2010). These compounds are relatively less efficacious and have not been tested in clinical studies. Finally, a structure-based virtual screening by Odolczyk et al. (2013) developed other small compounds that rescue F508del-CFTR functional cell surface expression by inhibiting the interaction of F508del-CFTR with keratin 8 (Colas et al., 2012).

Following HTS by Vertex Pharmaceuticals and chemical optimization, the most promising compound VX-809 (lumacaftor, VRT-826809) became the first corrector approved for clinical use as a combined oral treatment with VX-770 (trade name of combination Orkambi^®^). VX-809 has a higher potency and efficacy than VRT-325 and Corr-4a (Van Goor et al., 2010, 2011; Farinha et al., 2015). In F508del-homozygous HBE cells, VX-809 rescued total Cl^-^ secretion up to ∼14% of WT-HBE cells (Van Goor et al., 2011) and even up to ∼25%, as evaluated in HBE/HNE cells (Pranke et al., 2017). The VX-809 correction effect on F508del-CFTR is additive to VRT-325 and Corr-4a, suggesting a different mode of action (Van Goor et al., 2011). Interestingly, the correction effect of VX-809 can also be unmasked in HAE cells carrying only one copy of the F508del mutation: F508del/D1152H, F508del/394delTT, F508del/1717-1G > A (Pranke et al., 2017), F508del/G542X (Awatade et al., 2015; Pranke et al., 2017), F508del/Y1092X (Awatade et al., 2015), F508del/R117H, F508del/W1282X, or F508del/E60X (Gentzsch et al., 2016). Conversely, N1303K-CFTR, another Class II mutant, was not corrected by VX-809 in HBE cells, but R117H- (Gentzsch et al., 2016) and A561E-CFTR (Awatade et al., 2015) efficiently improved PM localization and function. The mechanism of action is not yet fully known; however, it has been shown that VX-809 stabilizes TMD1 (Loo et al., 2013; Okiyoneda et al., 2013), improves TMD1 folding (Ren et al., 2013), and stabilizes interdomain interactions between TMDs and NBDs (He et al., 2012; Loo and Clarke, 2017). Studies on *in vitro* liposomes and C18 analogs of VX-809 (Eckford et al., 2014) applying the alkyne-containing VX-809 derivatives (Sinha et al., 2015) have shown that VX-809 may bind to CFTR directly; however, the exact binding site has not been found. Evidence that VX-809 binds directly to CFTR is based on the precipitation of the VX-809-bound CFTR and on the visualization of VX-809-CFTR association in cells, thanks to the fact that the alkyne group of VX-809 derivatives can be conjugated to biotin-azide molecules through Cu’-catalyzed cycloaddition and by applying the conjugated biotin moiety.

As a whole, F508del-CFTR correctors in a single compound-treatment present modest effectiveness in CFTR rescue. It has been proposed that this limited efficacy is caused by the necessity to rescue multiple defects in F508del-CFTR at the same time (folding, activity, and stability defects) (Sloane and Rowe, 2010). This was illustrated by a clinical trial testing efficiency of VX-809 alone in F508del homozygote patients, which failed to demonstrate any improvement (Clancy et al., 2012).

Considering the unsatisfying results of the VX-809 corrector, Vertex Pharmaceuticals proposed to combine the potentiator (VX-770) and corrector (VX-809) because the *in vitro* analysis demonstrated that ivacaftor increased the open probability of F508del-CFTR by fivefold. Acute administration of VX-770 to VX-809-corrected primary HAE cells increased the F508del-CFTR function (Van Goor et al., 2011). However, later analysis of HBE cells showed a reduction in the correction efficacy of VX-809, as well as VX-661 when VX-770 was applied chronically (Cholon et al., 2014; Veit et al., 2014). Chronic co-treatment with VX-809 and VX-770 affected the folding efficiency of F508del-CFTR at the ER and its metabolic stability in Golgi apparatus and PM, reducing the F508del-CFTR density at the apical PM and function (Veit et al., 2014).

Initially, two Phase III clinical studies (TRAFFIC and TRANSPORT) were designed to assess the efficacy and safety of two different doses of VX-809 in combination with VX-770 in F508del-homozygous patients (Wainwright et al., 2015). Phase III clinical trials were shown to provide a benefit for patients. Patients over 12 years of age treated with the VX-809/VX-770 combination therapy for 24 weeks showed a mean absolute improvement in FEV_1_ between active treatment and placebo ranging from 2.6 to 4.0 percentage points (*P* < 0.001), a statistically significant weight gain, reduction in pulmonary exacerbations, and fewer hospitalizations. There were no major safety concerns. However, some increases in blood pressure and chest tightness/bronchospasm were reported (Wainwright et al., 2015). In 6–11-year-old patients, VX-770/VX-809 combination therapy demonstrated a statistically significant improvement in the lung clearance index (LCI) (-1.09 Unit; *p* < 0.0001), FEV_1_ (+2.4%; *p* = 0.02), sweat chloride (-21 mm/l; *p* < 0.0001) and body mass index *Z*-scores (+0.15, *p* < 0.0001) (Ratjen et al., 2017). Importantly, long-term follow up of patients on VX-770/VX-809 now show a slower decline in lung function over the study period compared with the rate of decline anticipated from registry data of patients not on VX-770/VX-809 (Konstan et al., 2017). Although the improvements seen with VX-770/VX-809 in F508del homozygotes were lower than those seen in VX-770 responsive mutations, the FDA and EMA approved this combination therapy (trade name Orkambi^®^) in 2015 for patients ages 12 and older who have two copies of the F508del mutation. In 2016, the FDA extended the license to patients aged 6–11 years and in August 2018 patients 2 years of age and older. However, it must be pointed out that clinical studies with Orkambi^®^showed a variable clinical responsiveness among patients. Less than 50% of patients had a FEV_1_ improvement by more than 5%, and only 25% of patients improved by more than 10% (Wainwright et al., 2015). This issue underlies the importance of the preclinical evaluation of CFTR modulators for each patient with the use of patient-specific biomarkers predictive of clinical efficacy.

A subsequent corrector developed by Vertex Pharmaceuticals—VX-661 (Tezacaftor)—has a structure similar to VX-809 but optimized pharmacokinetic properties. As reported by Van Goor et al., VX-661 increases Cl^-^ transport in F508del-homozygous HBE cells (from 2.5 to 8.1% of normal levels in WT-HBE) and in other additional CFTR mutant HBE cells (including mutations associated with gating defects and residual CFTR function) (Fidler et al., 2017). The combination of VX-661 and VX-770 increases Cl^-^ transport to 15.7% of normal Cl^-^ transport and improves ciliary beat frequency and fluid transport. Pranke et al. (2017) confirmed these results in HBE/HNE cells from F508del-homozygous patients and showed that Cl^-^ secretion increased up to 27.4% of normal WT cells. An increase of Cl^-^ transport was also measured in HBE cell cultures from heterozygous patients with genotypes F508del/394delTT and F508del/1717-1G > A (Pranke et al., 2017). After successful preclinical tests of VX-661, Vertex Pharmaceuticals next proposed a combination therapy of VX-661 and VX-770 originally for patients with two copies of the F508del mutation. Two separate multicenter clinical studies assessed the efficacy and safety of VX-661/VX-770 in patients 12 years of age and older: the EVOLVE study (Phase 3, randomized, double-blind, placebo-controlled, parallel group study) for patients homozygous for the F508del mutation; and the EXPAND study (Phase 3, randomized, double-blind, placebo-controlled, crossover study) for patients with one mutation that results in residual CFTR function in *trans* with the F508del mutation. The mean absolute improvement in FEV_1_ in the EVOLVE study was 4% from baseline for those treated with active compounds compared with the placebo. The rate of pulmonary exacerbation was 35% lower in the VX-661/VX-770 group than in the placebo group (Taylor-Cousar et al., 2017). In the EXPAND study, the combination treatment demonstrated a mean absolute improvement of 6.8% compared with the placebo. VX-770 alone improved FEV_1_ only by 4.7% compared with the placebo (Rowe et al., 2017). Additionally, VX-661/VX-770 did not induce the chest tightness or drug–drug interactions observed with Orkambi^®^. At the beginning of 2018, the FDA approved combination VX-661/VX-770 (trade name Symdeko^®^) to treat CF in people ages 12 and older who have two copies of the F508del mutation and those who have at least one residual function mutation from the following: A455E, E56K, R74W, A1067T, E193K, R117C, D110E, F1052V, R347H, D110H, F1074L, R352Q, D579G, K1060T, R1070W, D1152H, L206W, S945L, D1270N, P67L, or S977F; or one following splice mutations: 711+3A→G, 3272-26A→G, E831X, 2789+5G→A, or 3849+10kbC→T. A Phase III trial is ongoing to evaluate the safety and efficacy of Symdeko^®^in patients ages 6–11.

A study presented by Zawistoski et al. (2016) introduced a novel F508del-CFTR corrector—FDL169—whose potency and efficacy is comparable to VX-809. FDL169’s mechanism of action could possibly be similar to that of VX-809 because combining FDL169 and VX-809 does not further increase F508del-CFTR activity. Interestingly, the inhibitory effect of VX-770 on FDL169 activity is weaker than on VX-809. This new corrector could be an alternative for VX-809.

The Galapagos Company has developed novel correctors through HTS: GLPG2222 and GLPG2851 (C1). The chemical structure of GLPG2222 (Wang et al., 2018) is similar to the structures of VX-809 and VX-661; however, it was reported to be more potent. *In vitro* characterization demonstrated that GLPG2222 is highly functional in primary patient cells carrying two copies of the F508del mutation. The first one—GLPG2222—is currently being evaluated in a Phase II clinical study, whereas the second—GLPG2851—is currently in a Phase I study.

The modest efficacy of Orkambi^®^and Symdeko^®^triggered the development of next-generation drugs. Vertex Pharmaceuticals performed HTS on a cell model treated with VX-809 or VX-661 to search for more potent correctors, which yielded compounds that have additive rescue activity. VX-440, VX-152, VX-659, and VX-445 have been tested in separate Phase II clinical trials as a triple-combination therapy, together with VX-661 and VX-770, in adult CF patients carrying two copies of the F508del mutation or one copy of F508del and one copy of minimal CFTR function mutation. The initial results of the Phase II trials presented a significant increase in FEV_1_ for all groups of patients treated with the triple-combination therapy when compared with the placebo (up to 13.3% for VX-659 and 13.8% for VX-445), and a significant reduction in Cl^-^ levels in the sweat test. The triple-combinations of VX-661/VX-770 with VX-445 or VX-659 have been tested in Phase III trials (Davies et al., 2018; Keating et al., 2018). *In vitro* functional tests in F508del-homozygous HBE cell cultures demonstrated that the triple combination treatment of VX-661/VX-770/VX-152 improved CFTR activity up to ∼75% and VX-661/VX-770//VX-440 up to ∼67% of normal HBE cells. Subsequently, HBE cells with one copy of the F508del mutation were corrected up to ∼47% with the triple combination containing VX-152 and up to ∼43% with the triple combination including VX-440. Interestingly, an important correction over 50% of normal CFTR function was also observed in cells with genotypes “F508del/minimal function” on triple-combination regiments VX-661/VX-770VX-152, -VX-440, or -VX-659, with the highest improvement measured for VX-659.

Other next-generation correctors were introduced by Galapagos and Proteostasis Therapeutics. The initial results demonstrated that adding GLPG2737 to VX-809/VX-770 enhances the effectiveness of the treatment. The GLPG3221 (Galapagos) compound is under Phase I evaluation in healthy volunteers. Galapagos reported also that combinations of their first-generation correctors (GLPG2222 and GLPG2851) with their next-generation correctors (GLPG2737 and GLPG3221) and a potentiator GLPG1837 significantly increases Cl^-^ transport *in vitro* compared with the effect of Orkambi^®^. GLPG2222 passed early phase clinical trials and displayed improvement in potency and drug–drug interaction compared with VX-809 and VX-661 (Radar, 2016). GLPG2222 and GLPG2737 correctors together with the GLPG2451 potentiator are currently being tested in Phase II clinical trials as a triple-combination therapy (FALCON).

The PTI-801 (Proteostasis Therapeutics), a third-generation corrector, showed superior *in vitro* efficacy over known correctors and synergy. PTI-801 in triple combination together with Orkambi^®^and the PTI-428 amplifier is currently in a Phase I clinical evaluation of safety and tolerability. Initial positive results have been announced by Proteostasis company (Proteostasis Announces Positive Data from Ongoing Phase 1 Study of PTI-801 in Cystic Fibrosis Patients on Background Orkambi^®^Therapy, 2018). Proteostasis Company reported that potentiator PTI-808 enhanced the function of mutated F508del-CFTR *in vitro* and restored it to almost normal levels when combined with PTI-428 and PTI-801. This second triple therapy obtained fast-track status from the FDA.

Recently, Veit et al. (2018) identified new compounds—4172, 6258, 3151—stabilizing specific folding defects of F508del-CFTR. These rationally designed compounds lead to ∼50–100% of wild-type-level correction in immortalized and primary human airway epithelia and in mouse nasal epithelia. The strategy to use compounds that synergistically aim at distinct structural defects proved to be efficient to rescue mutant expression and function at the PM. Therefore, the combination of correctors could translate into improved clinical benefit in patients with CF.

### Amplifiers

Therapies to treat CF induced by mutations leading to decreased CFTR synthesis (Class V) requires agents that stimulate protein expression. The compounds enhancing the expression of CFTR protein, with a following increase of its quantity in the ER and the PM, are called amplifiers. Amplifiers could also be used as a therapy for other CFTR mutants when in combination with correctors and potentiators.

The PTI-428 compound from Proteostasis Therapeutics is a first-in-class CFTR amplifier that showed an *in vitro* increase in CFTR protein levels across genotypes. The amplifier could potentially improve mRNA stability and/or assist the processes surrounding CFTR transcription or translation. As reported by Molinski et al. (2017), PTI amplifier enhanced correction achieved with VX-809 and VX-770 treatment in CF cells and tissues from patients with rare CFTR mutations (ΔI1234_R1239-CFTR).

### Stabilizers

Class VI CFTR mutants and corrected Class II mutants, including F508del-CFTR localized to the PM present a reduced half-life because of increased endocytosis and decreased recycling. The instability of CFTR in the PM requires compounds that anchor mutant proteins in the membrane. Stabilizers are molecules that rectify the intrinsic protein instability and increase the CFTR residence time at the PM/decrease protein degradation rate from the PM.

It has been shown (Moniz et al., 2013) that hepatocytes growth factor (HGF) stimulated Rac1 signaling and contributed to F508del-CFTR anchoring at the cell surface through interactions with NHERF-1. Co-treatment of cells with HGF and lumacaftor improved the rescue of F508del-CFTR and stimulated CFTR stabilization at the apical membrane compared with lumacaftor treatment alone (Loureiro et al., 2015). An increase in CFTR interaction with NHERF-1 and subsequent stabilization of the CFTR mutant at the PM was also observed in airway cells treated with vasoactive intestinal peptide (VIP) (Rafferty et al., 2009).

Other strategies to stabilize CFTR at the PM by decreasing its endocytosis rate include cAMP signaling through EPAC1 (a guanine nucleotide exchange factor exchange protein directly activated by cAMP) (Lobo et al., 2016) and an inhibition of *S*-nitrosoglutathione reductase with *S*-nitrosylating agents, such as the endogenous *S*-nitrosoglutathione (GSNO). This latter strategy prevents CFTR interactions with Hsp70/Hsp90 chaperones (Marozkina et al., 2010; Zaman et al., 2016).

Cavosonstat (N91115, Nivalis)—an inhibitor of *S*-nitrosoglutathione reductase (GSNOR) through inhibiting GSNOR—increases *S*-nitrosoglutathione levels and leads to CFTR maturation and PM stability (Donaldson et al., 2017). A phase II clinical study is now being conducted to test Cavosonstat for patients with two copies of the F508del mutation in combination therapy with VX-809/VX-770 or for patients with gating mutants and receiving VX-770.

## Conclusion

New light has been shed on the molecular targets and pathways for therapeutic strategy thanks to the increasing comprehension of the cellular consequences of CFTR mutations.

The astonishing results of clinical trials with protein therapy demonstrate the clinical efficacy of mutation-personalized therapy. Nowadays, scientists are convinced that an improvement of CFTR function at the molecular level can translate into an improvement in lung function and significantly improve the daily life of the patients and, most likely, their survival. It is expected that the near future will herald an era when therapeutic options will be motivated by personalized information.

Future perspectives are to develop mutation-specific and mutation-independent therapies that achieve near wild-type processing and function, as in case of the triple-combination therapy for the F508del-CFTR mutant. Further studies, however, will be needed to assess long-term efficacy and tolerance. Importantly, DNA or mRNA editing in preclinical development may allow for correct non-rescuable mutations and ultra-rare genotypes that are not targeted by current protein therapies. The next challenge is to implement those therapies in newborns with the aim of targeting the basic defect that prevents organ injury in this population.

## Author Contributions

IP, AG, and IS-G wrote the manuscript. AH and AE reviewed the manuscript.

## Conflict of Interest Statement

IS-G is principal investigator for studies funded by Vertex Pharmaceuticals, has received research fundings from Vertex Pharmaceuticals, and is part of the scientific Advisory board of Proteostasis Inc. The remaining authors declare that the research was conducted in the absence of any commercial or financial relationships that could be construed as a potential conflict of interest.
